# Calix‐Like Metal–Organic Complex for High‐Sensitivity X‐Ray‐Induced Photochromism

**DOI:** 10.1002/advs.201500224

**Published:** 2015-11-19

**Authors:** Hao Zhang, Xintao Wu

**Affiliations:** ^1^ State Key Laboratory of Structural ChemistryFujian Institute of Research on the Structure of Matter Chinese Academy of SciencesFuzhouFujian350002P. R. China; ^2^ School of Chemistry and Chemical EngineeringUniversity of Chinese Academy of SciencesBeijing100049P. R. China

**Keywords:** electron transfer, inclusion compounds, metal complex, photochromism, X‐ray detection

## Abstract

Metal‐organic complexes (MOCs) as promising candidates for directly visual X‐ray detection at room temperature are rare and discovered unexpectedly, even though every crystalline material needs X‐ray diffraction studies. Here, we report a rational strategy of mimicking host‐guest system for developing high‐sensitive X‐ray‐induced photochromic materials. Two resulting calix‐like metal‐organic complexes (cMOC‐1 and cMOC‐2) were prepared by encapsulating the electron‐capturing “guest” into the cavity of calix‐like electron‐donating “host.” One of them (cMOC‐1) achieves instantaneous X‐ray‐induced photochromism and easy recovery by synergizing the aprotic matrix of MOC and similar host‐guest interaction. Their strikingly different response to X‐ray irradiation resulting from the structural difference demonstrates the feasibility and acceptability of our design strategy. This strategy may open new perspectives for developing high‐performance photo‐responsive functional materials.

Ionizing radiation especially for X‐ray has long been known to increase the risk of cancer which is yet used widely in medical diagnosis and treatment, airport security, radiological protection, and industrial crack detection.^1^ Besides, resent nuclear accidents such as the *Fukushima Daiichi* catastrophe has brought into focus the need to find effective strategies for the rapid and convenient detection of this carcinogen. Metal‐organic complex (MOC) used as a new type of X‐ray‐induced photochromic material has gained tremendous attention owing to its directly visual detection at room temperature. Although every crystalline material needs exposure to X‐ray irradiation during a single‐crystal X‐ray diffraction determination, only a few room‐temperature X‐ray‐induced photochromic MOCs have been reported which are less sensitive and discovered unexpectedly.^2^

The photochromic behavior needs the formation of photo‐induced bistable systems based on electron transfer (ET) process.^3^ X‐ray possesses a much higher threshold energy to determine the electronic dissociation, so enhancing the stability of the photo‐activated state is considered as the key factor in constructing the photo‐induced bistable systems of the X‐ray‐induced photochromic material. Serving as appropriate aprotic matrices, the increased dimensionality and structural robustness of MOC offers a unique platform to stabilize and confine the activated species for availably preventing the dimerization/oligomerization of photo‐activated radial cations.^4^ Meanwhile, a series of cucurbit[n]urils (CBn) and calixarene derivatives with selective inclusion of reactive species emerge to provide an effective alternative to this mission.^5^ We are greatly interested to design the novel calix‐like metal‐organic complex (cMOC) by mimicking the architecture of CBn and calixarene, because such material might combine the radical‐stabilized character of MOC with similar host‐guest interaction to give rise to the stability of the photo‐activated state in response to X‐ray irradiation. Furthermore, the thermodynamic and kinetic stability of MOC can have a considerable advantage over pH‐ and temperature‐sensitive hydrogen‐bonded systems.^6^

Considering that MOC allows for judicious choice of metal centers and organic linkers of various geometries and functionalities, we therefore designed a novel triazine‐based polycarboxyate ligand [piperazine‐*N*,*N*′‐bis(2,4,6‐triazinyl‐3,5‐bis(*N*,*N*′‐iminodiacetic acid))] (H_8_PTIA, **Figure** **1**c) which has multiple metal binding sites and hydrogen bonding modes to provide the probability of host‐guest interaction. Meanwhile, it is more extended and flexible than other triazine‐based ligands,^7^ hence preferring to form a ribbon‐like sheet then bent into the calix‐like shape by metal‐ligand orientation. More importantly, the ligand can serve as an excellent electron donor to generate the radical‐ion species. The in situ UV‐irradiated product shows a noticeable single‐peak radical electron paramagnetic resonance (EPR) signal at *g* = 2.0036 but no color change to the naked eye. We also investigated directly the similar property under X‐ray irradiation, but no further obvious change was observed (Figure S2, Supporting information). Therefore, the process of visible color change and X‐ray‐induced ET should need the participation of other factors.

**Figure 1:**
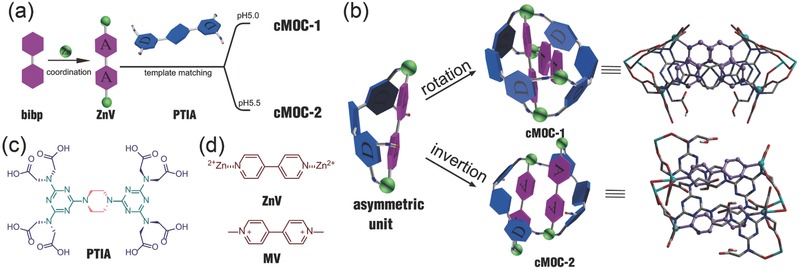
a) Synthetic route to obtain the cMOCs. b) Structural comparison between the calix‐like subunits of cMOC‐1 and cMOC‐2; gray: C; red: O; blue: N; cyan: Zn. c) Schematic representation of the H_8_PTIA ligand; red: flexible bent linker; blue: functional group unit; purple: metal‐binding sites. d) Chemical structures of ZnV and MV.

Usually, the reported photochromic MOCs incorporate viologen or its derivatives as the electron accepter,^8^ but the acute toxic methylviologen (MV) widely utilized as herbicides exercises great influence on the harmless application.^9^ The various coordination geometries of Zn ion facilitate us to coordinate directly it with 4,4′‐bipyridine (bibp) as structural substitution of MV (ZnV: zinc viologen) (Figure 1d).^10^ Besides, the Zn ion as d^10^ metal ion can exhibit photoactive capability.^11^

With partly deprotonated H_2_PTIA^6−^, bibp, and the Zn ion, we successfully prepared two 2D layer cMOCs: [Zn_3_(H_2_PTIA)(bibp)(H_2_O)_1.5_]·6H_2_O (cMOC‐1) and [Zn_3_(H_2_PTIA)(bibp)(H_2_O)_2_]·4.5H_2_O (cMOC‐2) which were crystallized in different space groups (*Pccn* in cMOC‐1 and *P*‐1 in cMOC‐2, respectively). Their phase purity is confirmed by powder X‐ray diffraction (PXRD) (Figures S3 and S4, Supporting Information). The pH‐dependent self‐assembly process of cMOCs can be described as shown in Figure 1a. Except the number of coordinated and lattice water molecules, the asymmetric units of both cMOCs have similar formula components, which both contain one independent Zn cation, one partly deprotonated H_2_PTIA ligand and one bibp ligand (Figure S5, Supporting Information). The bibp molecule coordinates firstly with the Zn cation to form ZnV acting then as a template. In the weak basic synthesis condition, the H_8_PTIA ligand was partly deprotonated to form six carboxyl groups and two uncoordinated carboxyl acids, which seem like dangling arms deviated from the ribbon‐like sheet. Interestingly, the template effect of ZnV directs all the arms and piperazine ring bending into the same side of the sheet to enfold ZnV like a semiring. The calix‐like subunit is constructed by the demerization of asymmetric units (Figure 1b). The striking structural difference between cMOC‐1 and cMOC‐2 derives from the different symmetry operation of asymmetric units. The subunit of cMOC‐1 (generated by the translation and rotation through a twofold screw axis) possesses a fascinating calix‐like shape in the cavity of which two ZnVs are decussately captured, while the parallel alignment of two ZnVs in the cMOC‐2 (generated by the translation and inversion through a inversion center) is so crowded that the subunit cannot form a perfect calix‐like cavity. Sharing the Zn metal nodes with neighboring subunits leads to a 2D layer. Then the 2D layers are packed together by weak intermolecular van der Waals interaction and aromatic interaction to form quite condensed 3D structure (Figure S6, Supporting Information). Additionally, the coordinated and lattice water molecules can form strong hydrogen‐bond interactions with the uncoordinated carboxylate oxygen atoms of the host framework to expand and stabilize the 3D architecture (Tables S1 and S2, Supporting Information).

cMOC‐1 and cMOC‐2 both exhibit similar X‐ray‐induced photochromic property, but show strikingly different response to X‐ray irradiation. MOC‐1 can undergo a rapid photochromic transformation from pale yellow to bluish violet upon X‐ray irradiation at room temperature. An activation analysis showed that coloration can be induced by hard X‐ray source (Mo Kα, *λ* = 0.71073 Å; powered at 4 kW; Figure S7, Supporting Information), soft X‐ray source (Cu Kα, *λ* = 1.54056 Å; powered at 4 kW and Al Kα, *λ* = 8.357 Å; powered at 150 W). Such a process is rather sensitive to Al Kα radiation and can even be fulfilled immediately upon X‐ray irradiation. Comparing to the instantaneous response of cMOC‐1, cMOC‐2 needs about 30 s to change color obviously (Figure S8, Supporting Information). Selecting lower exciting energy of X‐ray source can amplify and highlight the comparison of the visual color change, so a Cu Kα radiation (*λ* = 1.5418 Å) in the CCD diffractometer equipped with an in situ visualization system facilitates our evaluation as shown in **Figure** **2**. Clearly, the color became much deeper with increasing exposure time, and cMOC‐1 is much more sensitive than cMOC‐2. The violet samples (cMOC‐1a and cMOC‐2a) are stable in air in a dark room for 2 days at ambient temperature followed by a slow reversion back to original color over 1 week in a refrigerator (−10 °C). They can be easily decolored by heating at 80 °C for less than 10 min in air or in an argon atmosphere, whereas other reported X‐ray‐induced photochromic systems need higher temperature and rigorous condition to be recovered.^2^ This reversible photochromic transformation can repeat for more than ten cycles by alternated X‐ray irradiation and heating, and no noticeable change of photochromic properties is observed. Both PXRD and IR analyses showed that both cMOCs remained the same crystal structure and organic components through the reversible photochromic transformation (Figures S3, S4, and S9, Supporting Information).

**Figure 2:**
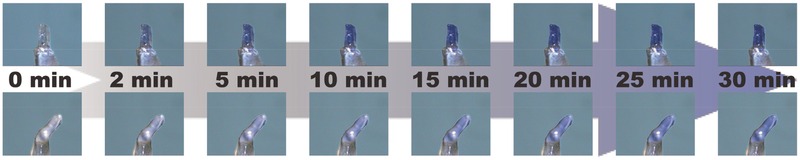
The X‐ray‐induced photochromic process irradiated by Cu Kα source. The process of cMOC‐1 (top) and cMOC‐2 (bottom) recorded in a CCD diffractometer (Cu Kα, *λ* = 1.54056 Å; powered at 4 kW) equipped with an in situ visualization system.

To indicate that the photochromism is due to an intrasolid‐state transformation, we sealed the original sample under vacuum which then also generated X‐ray‐induced photochromism and heated recovery. Similar to the previous reports,^2^ this color change is caused by the generation of reduced ZnV radical cations. As shown in **Figure** **3**a, the original samples exhibit no EPR signal, but a noticeable single‐peak radical signal is observed and increased with prolonged X‐ray irradiation time. The relative EPR signal intensity of cMOC‐1a is higher for its stronger response to X‐ray irradiation as observed above (Figure 3b). Meanwhile, the UV‐vis spectra show that the photo‐activated state produces a new absorption band at the region from 450 to 600 nm which informs that the ET absorption process existed upon X‐ray irradiation (Figure S10, Supporting Information). Furthermore, the in situ XPS measurement also supports the X‐ray‐induced ET behavior. As shown in Figure 3c, the core‐level spectra of both Zn 2p and C 1s are almost identical before and after X‐ray irradiation. The N 1s core‐level spectra can be fitted by two peaks at the binding energy of 399.1 and 400.6 eV, respectively. After in situ X‐ray irradiation, a prominent shift toward a lower N 1s binding energy occurs and a new signal appearing at 399.5 eV corresponds to the nitrogen atom of the tertiary amino and pyridyl radicals which both prove that the ZnV moiety is an electron accepter (Figure S11, Supporting Information). Although the O 1s spectra could not be separated successfully by peak‐resolution techniques for its complicated bonding environment, the O 1s binding energy of cMOC‐1a is 0.6 eV higher than that of cMOC‐1 due to the electron dissociation of the carboxyl oxygen atoms. Similar changes of the N 1s and O 1s XPS core‐level spectra can be observed between cMOC‐2 and cMOC‐2a under identical condition (Figure S12, Supporting Information).

**Figure 3:**
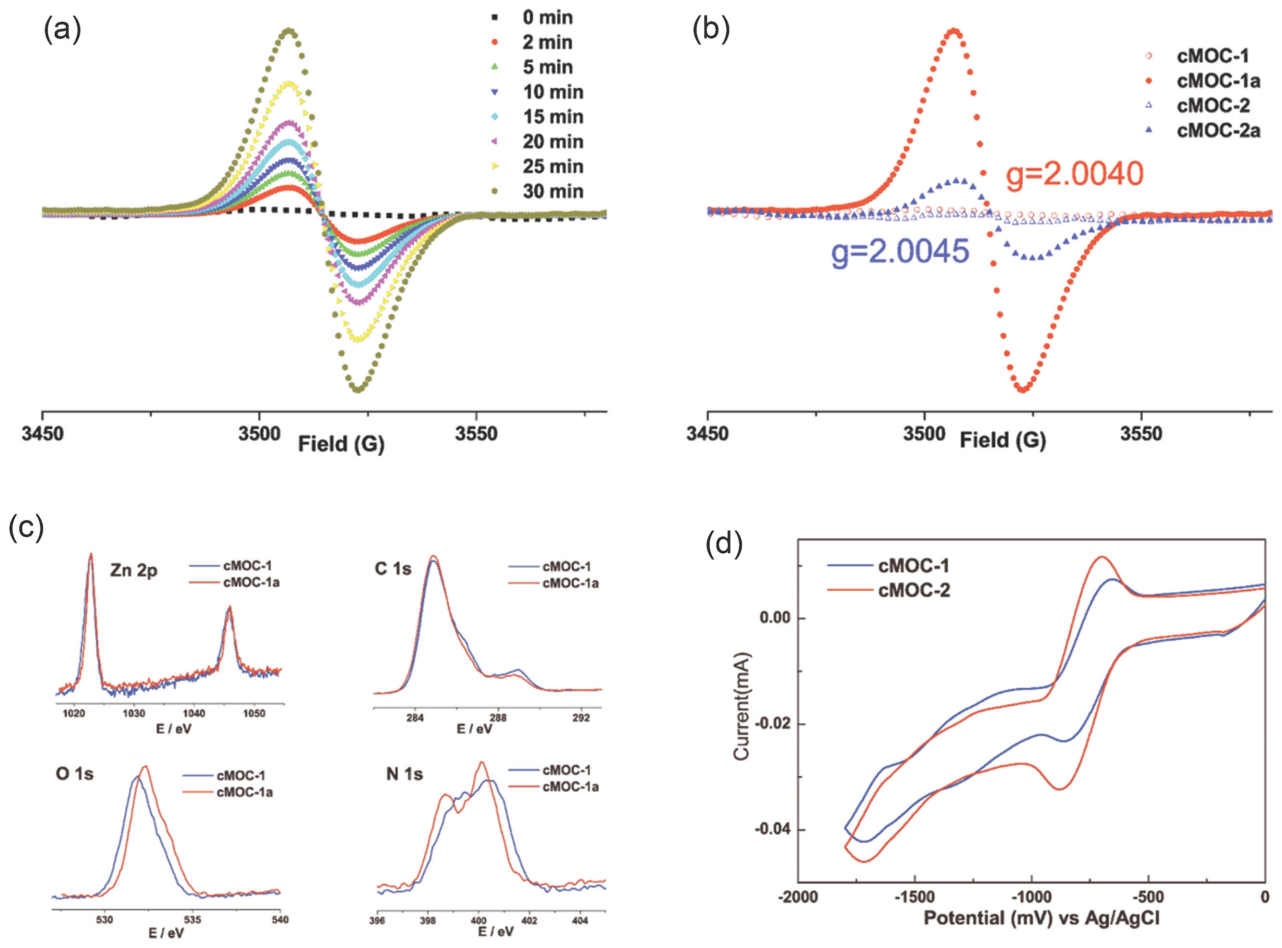
a) The time‐dependent EPR spectra of cMOC‐1 upon X‐ray irradiation. b) The comparison of EPR signal intensity between cMOC‐1a and cMOC‐2a. c) In situ XPS core‐level spectral differences of cMOC‐1 and X‐ray irradiated cMOC‐1a. d) Solid‐state cyclic voltammetric curves of cMOC‐1 and cMOC‐2 in a 0.1 m [(*n*‐Bu)_4_N]PF_6_/MeCN electrolyte.

It should be noted that no obvious color change is observed for both cMOCs upon UV irradiation for 24 h, which is also verified by the UV‐vis and EPR spectral change (Figures S13 and S14, Supporting Information). As shown in **Figure** **4**a, n‐π^∗^ conjugation is weakened to raise the highest occupied molecular orbital (HOMO) energy and lower the lowest unoccupied molecular orbital (LUMO)energy for the coplanar conformation of the melamine core is destroyed by template matching. Hence, the PTIA sector (the coordinated H_8_PTIA unit in the cMOCs) requires much higher exciting energy to obtain PET excited state with respect to the free ligand which can make response to UV irradiation. As discussed above, cMOC‐1 and cMOC‐2 have almost the same formula component, but show significant difference of susceptibility to X‐ray irradiation which can be mainly attributed to their completely different structural features. Considering that cMOC‐1 has more perfect calix‐like subunit, the architecture may play an important role in the stabilization of photo‐activated species just like host‐guest system. In the imitating system, the calix‐like ring mainly constructed by two PTIA sectors can be regarded as “host,” while two ZnVs captured in the cavity can be considered as “guest.” The similar role may be supposed to depend on the following structural reasons: (i) the ZnV radical anions are captured and stabilized in the calix‐like cavity by delocalization of the unpaired electron between host and guest through metal coordination. The shorter ET pathway of cMOC‐1 can not only strengthen metal‐assisted ligand‐to‐ligand charge transfer (LLCT) interaction to show faster response to X‐ray irradiation and easy recovery, but also favor the stabilization and delocalization of the activated ZnV radical anions (Figure 4b,c); (ii) compared to dihedral angle (42.202°) between two pyridyl rings of ZnV in the cMOC‐2, two approximate coplanar pyridyl rings (11.970°) in the cMOC‐1 show stronger π‐π conjugation to stabilize the radical anions (Figure S6, Supporting Information). The pale yellow color of cMOC‐1 crystal may be ascribed to the coplanar structural character with respect to the colorless crystal of cMOC‐2. Furthermore, the electrochemical behaviors were also investigated in line with the above structure‐property relationship. The half‐wave potential (*E*_1/2_) of cMOC‐1 is obviously shifted to more positive value compared to cMOC‐2, reflecting the reduced state cMOC‐1a is easier to be obtained. It indicates that the stronger stabilization of the photo‐activated reduced state can be offered by the perfect calix‐like architecture in the cMOC‐1. The reported X‐ray‐induced photochromic materials NTHU‐9‐MV and JU99 can on the other hand provide corroborative evidences to the advantage of the similar role (Figure S15, Supporting Information). These results all demonstrate that our mimic motive to stabilize the photo‐activated state comes true by constructing cMOC analogous to the architecture of CBn and calixarene.

**Figure 4:**
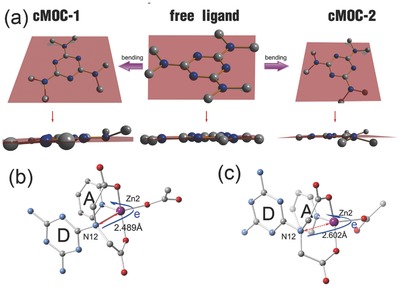
a) View of the different conformations of the melamine core in the free PTIA ligand and PTIA sectors of cMOC‐1 and cMOC‐2. The light red planes represent the ideal coplanar conformation of the melamine core. The ET pathway of metal‐assisted LLCT illustrated by blue arrow in the b) cMOC‐1 and c) cMOC‐2.

In summary, we have illustrated a judicious design strategy to construct two novel X‐ray‐induced photochromic materials by mimicking supramolecular constructs. The strategy can synergize the radical‐stabilized character of the crystalline MOC and similar host‐guest interaction to stabilize the activated radical, moreover their structure‐property analysis demonstrates that the calix‐like architecture can adjust and optimize metal‐assisted LLCT to achieve instantaneous response to X‐ray irradiation at room temperature and easy recovery under mild condition. We also investigate the electronic and optical properties to elucidate the nature of the charge transfer behavior which results in the reversible photochromic behavior regulated by X‐ray irradiation. We believe that this novel approach opens a new perspective for the development of high‐performance X‐ray‐responsive functional materials.

## Experimental Section

*Synthesis of cMOC‐1*: Zn(NO_3_)_2_·6H_2_O (178.5 mg, 0.6 mmol) and 4,4′‐bipyridine (31.2 mg, 0.2 mmol) were dissolved into water (5 mL) and stirred for 2 h. The transparent solution was then added into the H_8_PTIA aqueous solution (153.6 mg, 0.2 mmol in 5 mL of water), which was firstly adjusted to an acidity of pH ≈ 5.0 with 1 m NaOH aqueous solution. After stirred for 10 min, the mixture was then transferred to and sealed in a Teflon reactor (20 mL) and heated at 140 °C for 72 h. After that the mixture was cooled to 30 °C at about 5 °C h^−1^. Pale yellow prismatic crystals were obtained (65.8% yield based on H_8_PTIA). EA (%) calculated for cMOC‐1: C, 34.59; H, 3.95; N, 15.69; found: C, 34.68; H, 3.62; N, 15.88.

*Synthesis of cMOC‐2*: cMOC‐2 can be obtained by the same synthetic procedures as cMOC‐1 except that the H_8_PTIA aqueous solution was firstly adjusted to pH ≈ 5.5 with 1 m NaOH aqueous solution. Colorless schistose crystal of cMOC‐2 were obtained (61.2% yield based on H_8_PTIA). EA (%) calculated for C_36_H_47_N_14_O_22.50_Zn_3_: C, 35.09; H, 3.84; N, 15.92; found: C, 34.79; H, 3.90; N, 15.88.

*Crystal Data for cMOC‐1*: C_36_H_49_N_14_O_23.50_Zn_3_, *M*_r_ = 1250.00, orthorhombic, space group *Pccn*, *a* = 27.087(6) Å, *b* = 17.270(4) Å, *c* = 21.169(5)Å, *α* = *β* = *γ* = 90.00°, *V* = 9903(4) Å^3^, *Z* = 8, *ρ*_calcd_ = 1.677 mg cm^−3^, final *R*1 = 0.0766, w*R*2 = 0.2325 (*R*_int_ = 0.0632), GOF = 1.011; cMOC‐2: C_36_H_47_N_14_O_22.50_Zn_3_, *M*_r_ = 1231.99, triclinic, space group *P*‐1, *a* = 12.906(7) Å, *b* = 13.998(8) Å, *c* = 14.716(9) Å, *α* = 109.490(5)°,*β* = 105.830(4)°,*γ* = 95.636(7)°, *V* = 2358(2) Å^3^, *Z* = 2, *ρ*_calcd_ = 1.735 mg cm^−3^, final *R*1 = 0.0853, w*R*2 = 0.2314 (*R*_int_ = 0.0519), GOF = 0.988.

CCDC 1405129 (cMOC‐1) and 1405128 (cMOC‐2) contain the supplementary crystallographic data for this paper. These data can be obtained free of charge from The Cambridge Crystallographic Data Centre via www.ccdc.cam.ac.uk/data_request/cif.

## Supporting information

As a service to our authors and readers, this journal provides supporting information supplied by the authors. Such materials are peer reviewed and may be re‐organized for online delivery, but are not copy‐edited or typeset. Technical support issues arising from supporting information (other than missing files) should be addressed to the authors.

Supplementary
