# Supplementary material for: Calix‐Like Metal–Organic Complex for High‐Sensitivity X‐Ray‐Induced Photochromism
Source: Adv Sci (Weinh). 2015 Nov 19;3(4):1500224. doi: 10.1002/advs.201500224 (PMC5063167; doi:10.1002/advs.201500224)
Supplement: Supplementary file 1 — Supplementary [file ADVS-3-0e-s001.pdf]

Copyright WILEY-VCH Verlag GmbH & Co. KGaA, 69469 Weinheim, Germany, 2015.

ADVANCED SCIENCE
Open Access

Supporting Information

for *Adv. Sci.*, DOI: 10.1002/advs. 201500224

Calix-Like Metal-Organic Complex for High-Sensitive X-Ray-Induced Photochromism

*Hao Zhang and Xintao Wu**

Copyright WILEY-VCH Verlag GmbH & Co. KGaA, 69469 Weinheim, Germany, 2013.

Supporting Information

Calix-like Metal-Organic Complex for High-Sensitive X-ray-Induced Photochromism

*Hao Zhang(s), and Xintao Wu(s)**

Experimental Section

Materials and Measurements. All the reagents and solvents for the syntheses were purchased from commercial sources and used as received, except H_8PTIA. All the measurements except for the elemental analyses were carried out in air at room temperature. Elemental analyses (C, H, and N) were performed on an Elementar Vario MICRO CHNOS elemental analyzer. The infrared spectra (IR) with KBr pellets were recorded in the range of 4000 – 400 cm^{-1} on a Perkin-Elmer Spectrum One FT-IR spectrometer. Thermal analyses (TGA) were performed on a TGA/DSC 1 $STAR^{e}$ system from room temperature to 1000 °C with a heating rate of 10 K/min under nitrogen. The ESI-MS spectrum was obtained from DECAX-30000 LCQ Deca XP mass spectrometer. The 1H NMR spectrum was recorded at 400 MHz on a Bruker Avance III NMR spectrometer. Powder X-ray diffraction (PXRD) data were collected on on a Rigaku Desktop MiniFlex 600 diffractometer using Cu-Kα radiation (λ = 1.54056 Å) powered at 600 W, and using the quartz plate as a sample holder. Solid state UV/Vis spectra were measured on a PerkinElmer Lambda-9500 UV/Vis/NIR spectrophotometer equipped with an integrating sphere, and using dry $BaSO_4$ as a ‘background’ matrix. Electron Paramagnetic Resonance (EPR) spectra with a laboratory UV light (365 nm, powered by 5 W) were recorded on a Bruker ELEXSYS E500 EPR spectrometer with a 100 kHz magnetic field modulation. X-ray photoelectron spectroscopy (XPS) studies were performed in a ThermoFisher ESCALAB 250Xi system, and the C 1s line at 284.8 eV was used as the binding energy reference.

Synthesis of the H_8PTIA ligand. As shown in Figure S1, Cyanuric chloride (3.73 g, 0.02 mol) was added to 50 ml H_2O and then was stirred at 0 °C for 30 min. After that a solution of iminodiacetic acid (5.43 g, 0.04 mol) and NaOH (4.8 g, 0.12 mol) in water (40 ml) was added dropwise into the above solution and stirred at < 5 °C for 1.5 h. Then the reaction mixture was allowed to warm to room temperature. After stirring at room temperature for 3 h, 1,4-piperazine (0.87 g, 0.01 mol) sodium hydroxide solution was added and the mixture was heated slowly and refluxed for 6 h. During this period, the pH of the mixture was maintained between 10 and 11 by the addition of sodium hydroxide solution. After cooling, the solution was acidified with concentrated hydrochloric acid whereupon a crystalline solid precipitated out. The mixture was allowed to cool to room temperature and filtered. After washing thoroughly with cold water, the product was dried in a vacuum oven at 60 °C and the yield was about 90%. 1H NMR (D_2O+NaOD, 400 MHz): δ3.62 (s, 8H), 4.00 (d, J=14.3 Hz, 16H). 13C NMR (D_2O+NaOD): δ 43.20, 51.82, 166.34, 178.66. ESI-MS: m/z 767.5 ($[M–H]^{-}$, 100%). Elemental Anal. Calculated for H_8PTIA ($C_{26}H_{32}N_{12}O_{16}$, %): C 40.63, H 4.20, N 21.87; found: C 40.74, H 4.18, N 21.85.

Single-Crystal X-ray Data Collection and Structure Determination. The single-crystal diffraction data of cMOC-1 and cMOC-2 was collected on a Rigaku Saturn 724HG CCD diffractometer equipped with graphite-monochromated Mo-Kα radiation (λ = 0.71073 Å). The collected data were reduced using the program CrystalClear (Rigaku and MSC, 1999) and an empirical absorption correction (multiscan) was applied. The structure of cMOC-1 and cMOC-2 was solved by direct methods and refined by the full-matrix least squares on F^2 using the SHELXTL-97 program.[1] All non-hydrogen atoms were refined with anisotropic displacement parameters. Hydrogen atoms attached to carbon were placed in geometrically idealized positions and included as riding atoms (C-H bond fixed at 0.97 Å); the H atoms on lattice water molecules are not located for high disorder and those on μ_2-OH_2 groups were located in a difference Fourier map and refined with O-H distance restraints of 0.85 Å. More details on the crystallographic studies as well as atomic displacement parameters are given in Supporting Data as CIF files.

Structural Description. The crystal structure was determined by X-ray crystallography at room temperature, revealing that cMOC-1 was crystallized in the orthorhombic space group *P*ccn. As shown in Figure S5, the asymmetric unit contains three Zn(Ⅱ) ion, one H_2PTIA^{6-} ligand, one bibp and one and a half coordinated water molecules. The three crystallographically independent Zn atoms exhibit different coordinated environments. The Zn1 ion is six-coordinated by four carboxyl oxygen atoms of two H_2PTIA^{6-} ligands [Zn – O = 2.027(4) – 2.136(4) Å] and two oxygen atoms of the coordinated water molecule [Zn – O = 2.093(4), 2.210(19) Å] to furnish a distorted [ZnO6] octahedron geometry, while the Zn2 ion is five-coordinated by one nitrogen atom of bibp [Zn – N = 2.022(5) Å], one nitrogen atom from the -$N(CH_2CO^{2-})_2$ group of the H_2PTIA^{6-} ligand [Zn – N = 2.489(5) Å], and three carboxyl oxygen atoms of two H_2PTIA^{6-} ligands [Zn – O = 1.948(4), 1.960(4), 2.005(4) Å], resulting in a distorted square pyramidal geometry. The Zn3 ion resides in a distorted tetrahedral coordination geometry defined by one nitrogen atoms of bibp [Zn – N = 2.018(5) Å] and three carboxyl oxygen atoms of two H_2PTIA^{6-} ligands [Zn – O = 1.942(4), 1.948(4), 1.955(4) Å]. cMOC-2 contains only more half one coordinated water molecule than cMOC-1 with similar formula component of the asymmetric unit, however it crystallized in the triclinic space group *P*-1. The only different coordination environment of the Zn(Ⅱ) ion between cMOC-1 and cMOC-2 is that the Zn2 ion becomes four-coordinated in a distorted tetrahedral coordination geometry on account of the longer distance between the Zn atom and the nitrogen atom of the flexible -$N(CH_2CO^{2-})_2$ group [Zn – N = 2.601(5) Å]. Similarly to cMOC-1, all the Zn–O, Zn–N distances are in the range of 1.953(5) - 2.237(5) and 2.030(6) - 2.048(6) Å, respectively.

Solid State Electrochemistry. Cyclic voltammograms (CVs) measurements were carried out using an AMETEK VersaSTAT 3 electrochemistry workstation. A three-electrode cell incorporating a CHI104 (CH Instrument, Inc. U. S. A.) glassy carbon working electrode, platinum wire auxiliary electrode and silver wire quasi-reference electrode was employed. The crystals were coated onto the surface of the working electrode by dipping the electrode in the slurry of the solid with acetone. Scans were performed in a 0.1 M tetrabutylammonium hexafluorophosphate $[(n\text{-}Bu)_4N]PF_6/CH_3CN$ electrolyte under flowing Ar.[2]

Study of X-ray-induced Photochromism. A Rigaku Desktop MiniFlexII diffractometer (Cu-Kα, λ = 1.54056 Å; powered at 450 W; spot size of irradiation: 10 mm in width) was used to prepare the bulky activated samples cMOC-1a and cMOC-2a for measurement and characterization. Hard X-ray source came from the above mentioned single-crystal XRD system, while the above XPS measurement system and SuperNova (Dual source) Diffractome (Cu-Kα, λ = 1.54184 Å; powered at 4 kW) were used as soft X-ray source. An *in situ* visualization system equipped in the source facilitates the evaluation of the exposure time for visual coloration. UV irradiation was performed with a YUHUA ZF-20D laboratory UV light (365 nm, powered by 12 W).

Figure S1: Synthetic Route to Obtain the H_8PTIA ligand

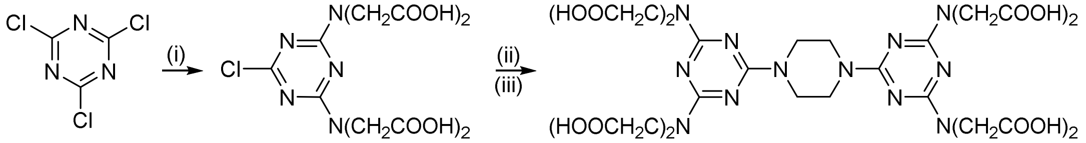

(i) 2 eq. iminodiacetic acid, NaOH, H_2O, 273 K-298 K; (ii) 0.5 eq. piperazine anhydrous, NaOH, H_2O, 453 K; (iii) HCl.

Figure S2: The photoinduced electron transfer (PET) of the H_8PTIA.

a) EPR spectra of PTIA and UV irradiated PTIA (PTIA-UV) for 10 minutes. b) EPR spectra of PTIA and X-ray irradiated PTIA (PTIA-X) for 6 hours.

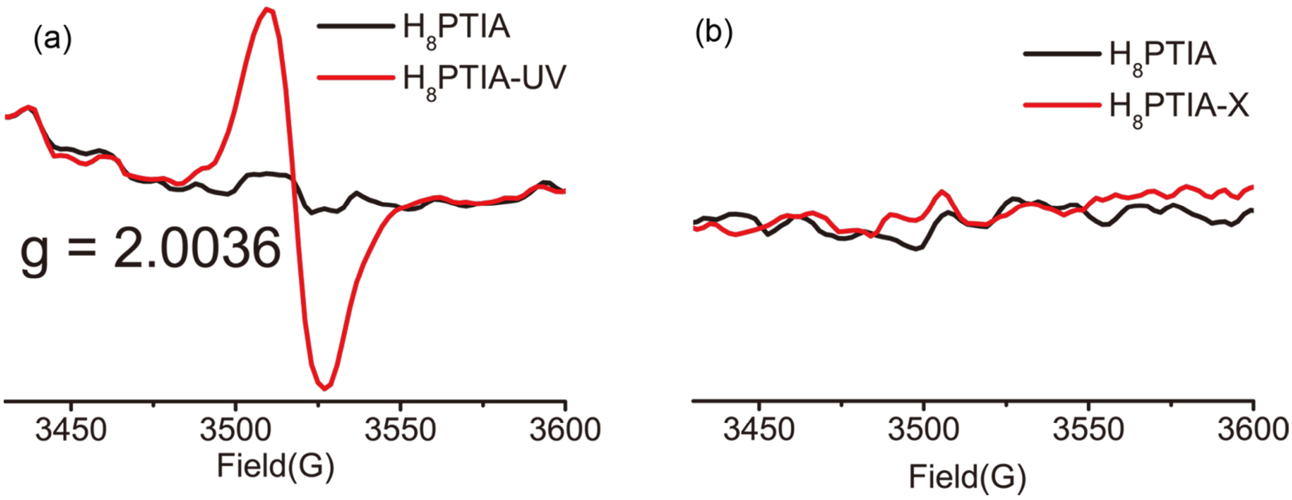

Figure S3: PXRD patterns for cMOC-1.
Black: The simulated PXRD curve is based on the single-crystal X-ray diffraction data of cMOC-1; red: as-synthesized cMOC-1; blue: X-ray irradiated cMOC-1a for 2 hours (cMOC-1a); dark cyan: the decolored cMOC-1a sample by heating at 80 °C for 10 min (cMOC-1a-decolored); magenta: cMOC-1 annealed at 200 °C for 1 h (cMOC-1-200 °C); dark yellow: cMOC-2 repeated for 10 cycles by alternated X-ray irradiation and heating.

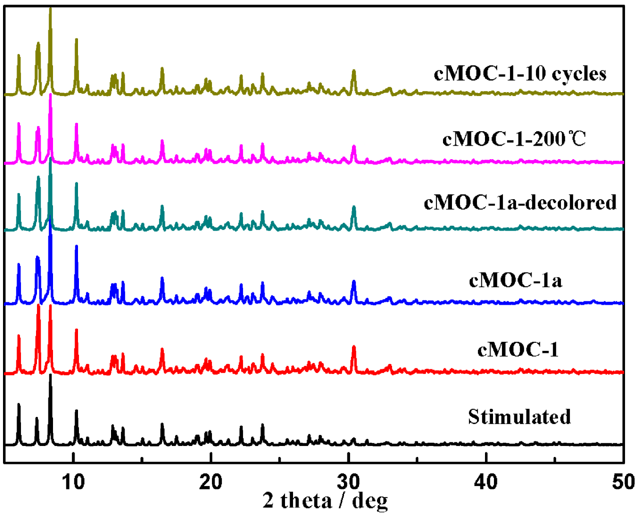

Figure S4: PXRD patterns for cMOC-2.
Black: The simulated PXRD curve is based on the single-crystal X-ray diffraction data of cMOC-2; red: as-synthesized cMOC-2; blue: X-ray irradiated cMOC-2a for 2 hours (cMOC-2a); dark cyan: the decolored

cMOC-2a sample by heating at 80 °C for 10 min (cMOC-2a-decolored); magenta: cMOC-2 annealed at 200 °C for 1 h (cMOC-2-200 °C); dark yellow: cMOC-2 repeated for 10 cycles by alternated X-ray irradiation and heating (cMOC-2-10cycles).

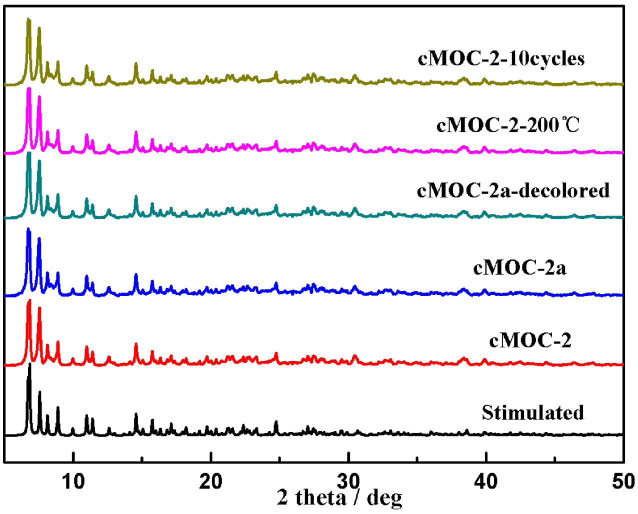

Figure S5: Coordination environment of the Zn centers in the asymmetric unit of cMOC-1 (left) and cMOC-2 (right).

Red elliptical ring shows that the different coordination environment of the Zn2 atom between cMOC-1 and cMOC-2.

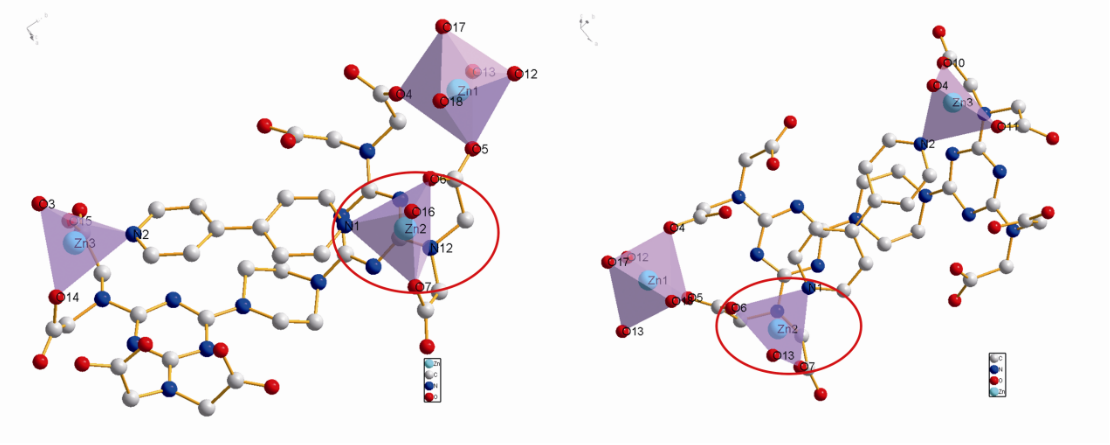

Figure S6: Packing diagram of cMOC-1 (a) and cMOC-2 (b).

It shows that the 2D layers are packed together to form quite condensed 3D structure. The pyridyl rings of two ZnVs in the cavity of every subunit are both twisted along the pivotal 1,1'-bond with the same dihedral

angles (11.970 ° and 42.202 ° respectively in the cMOC-1 and cMOC-2).

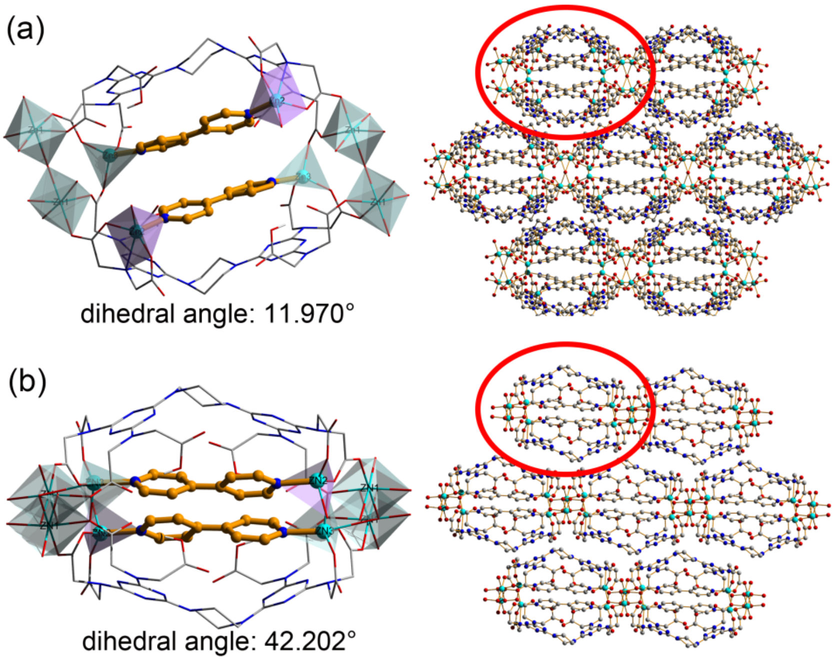

Figure S7: The X-ray-induced photochromic process irradiated by Mo-Kα source.
The process was recorded under X-ray irradiation for 1 h in a CCD diffractometer (Mo-Kα, λ =0.71073 Å; powered at 4 kW) equipped with an *in situ* visualization system.

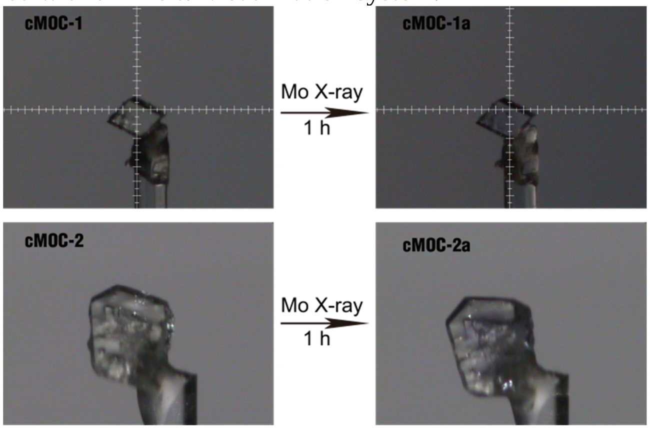

Figure S8: The X-ray-induced photochromic process irradiated by Al-Kα source.
The process of cMOC-1(top) and cMOC-2 (bottom) recorded in an X-ray photoelectron spectrometer (Al-Kα, λ=8.357 Å; powered at 150W) using a tablet (1 mm in thickness) of powdered single crystals. Red frame shows that cMOC-1 achieves instantaneous X-ray-induced photochromism powdered by Al-Kα source.

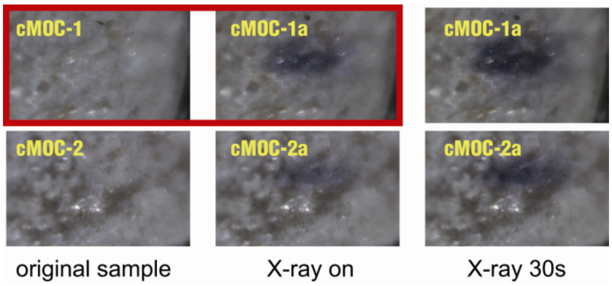

Figure S9: IR spectra of H_8PTIA, cMOC-1 and cMOC-2 before and after X-ray irradiation for 2h.

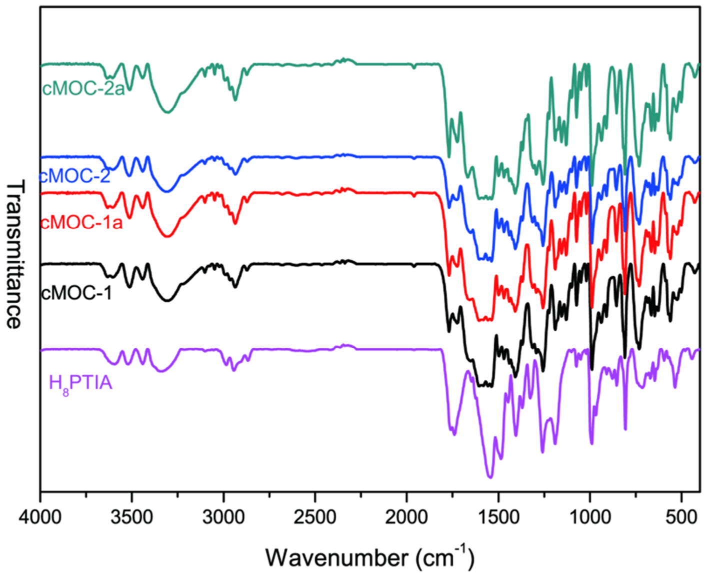

Figure S10: Photochromism and UV/Vis spectra for cMOCs and their activated sample.
(a) Photochromism and UV/Vis spectra for cMOC-1 (black) and X-ray irradiated cMOC-1a (red) for 2 hours. (b) Photochromism and UV/Vis spectra for cMOC-2 (black) and X-ray irradiated cMOC-2a (red) for 2 hours.

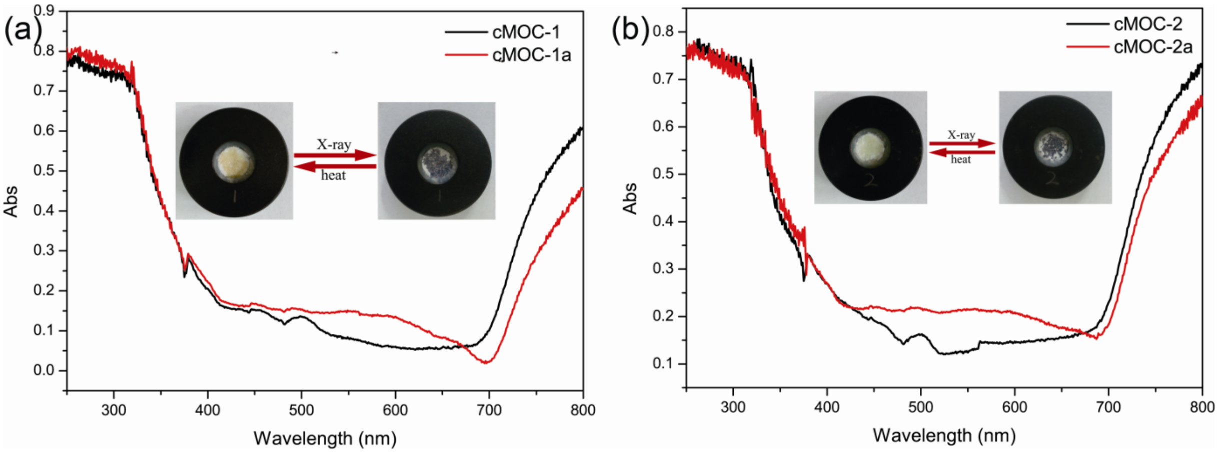

Figure S11: Resolved N 1s XPS (Al-Kα) core-level spectra of cMOC-1.
The spectra were obtained before (a) and after (b) *In situ* X-ray irradiation for 10min.

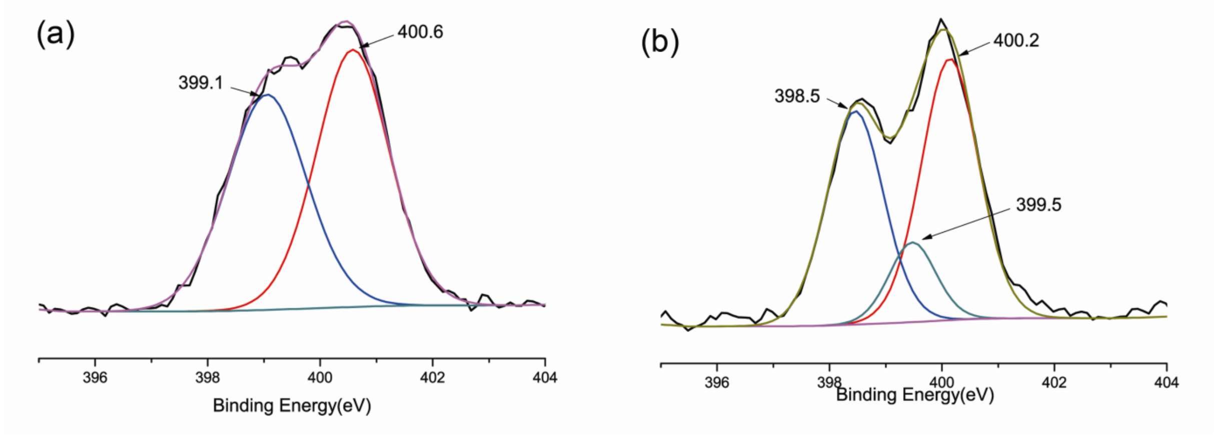

Figure S12: *In situ* XPS core-level spectral differences of cMOC-2 and X-ray irradiated cMOC-2a.

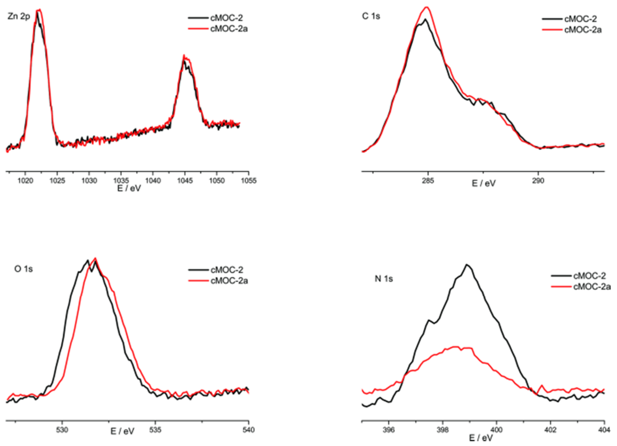

Figure S13: The comparison before and after UV irradiation for 24h in the cMOC-1 and cMOC-2.

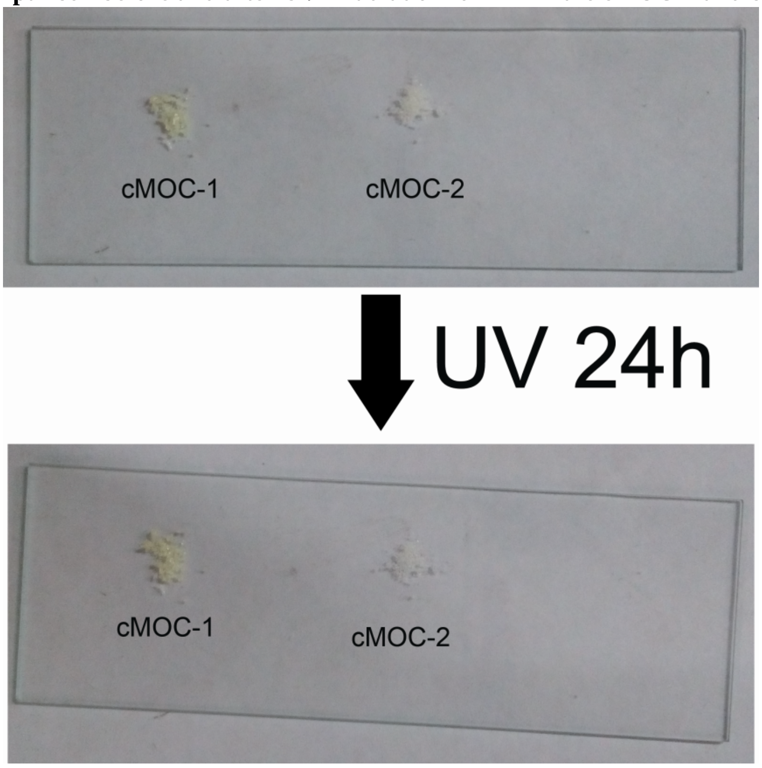

Figure S14: UV/Vis spectral change upon UV irradiation of cMOC-1 (a) and cMOC-2 (b) for 24h. Inset: The EPR spectral comparison of cMOC-1 and cMOC-2 before and after UV irradiation for 24 h.

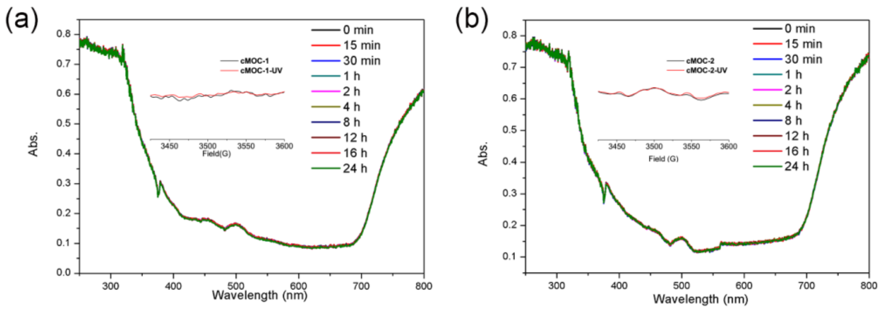

Figure S15: structures of NTHU-9-MV(left) and JU99(right) [3,4].

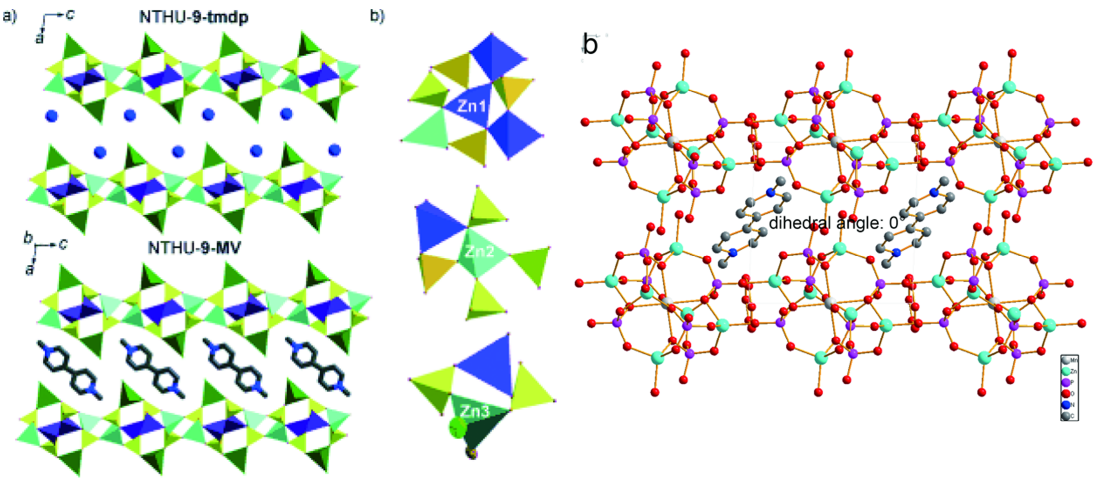

Figure S16: The TGA and DSC curves for cMOC-1.

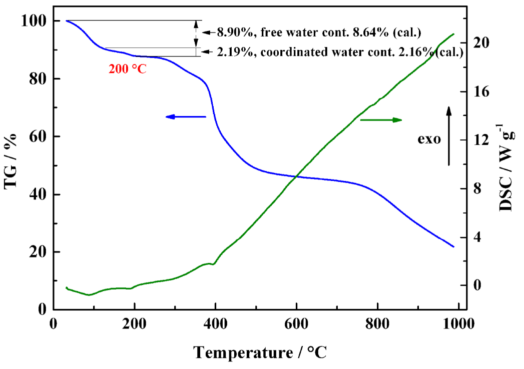

Figure S17: The TGA and DSC curves for cMOC-2.

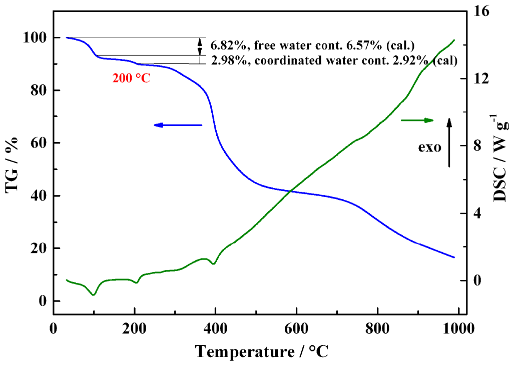

Table S1. The geometrical parameters of selected hydrogen bonds (Å, °) in the cMOC-1 frameworks.

D—H···*A*	*D*—H	H···*A*	*D*···*A*	*D*—H···*A*
O2—H2*A*···O9^{i}	0.85	2.34	2.992 (16)	134
O9—H9*A*···O11	0.85	1.68	2.507 (8)	164
O9—H9*A*···N13	0.85	2.38	2.814 (8)	112
O17—H17*A*···O14^{ii}	0.85	1.84	2.646 (5)	158
O18—H18*B*···O12^{ii}	0.85	1.94	2.788 (6)	174
O18—H18*C*···O2*W*	0.85	2.17	3.010 (11)	171

Symmetry codes: (i) *x*, −*y*+1/2, *z*−1/2; (ii) −*x*+1/2, −*y*+1/2, *z*.

Table S2. The geometrical parameters of selected hydrogen bonds (Å, °) in the cMOC-2 frameworks.

D—H···*A*	*D*—H	H···*A*	*D*···*A*	*D*—H···*A*
O2—H2*B*···O3	0.82	1.83	2.638 (8)	169
O2—H2*B*···N11	0.82	2.46	2.877 (8)	113
O15—H15*C*···O14	0.82	1.86	2.672 (7)	168
O15—H15*C*···N14	0.82	2.43	2.851 (8)	113
O17—H17*B*···O11^{i}	0.85 (1)	1.89 (2)	2.731 (6)	171 (12)
O17—H17*B*···O12^{i}	0.85 (1)	2.44 (11)	2.971 (6)	121 (10)
O17—H17*C*···O7^{ii}	0.86 (1)	1.84 (5)	2.638 (7)	155 (12)
O18—H18*B*···O10^{iii}	0.85 (1)	1.88 (7)	2.654 (7)	151 (13)
O18—H18*C*···O6	0.85 (1)	1.96 (6)	2.731 (6)	151 (11)

Symmetry codes: (i) $x-1$, $y-1$, $z-1$; (ii) $-x+1$, $-y$, $-z$; (iii) $-x+1$, $-y+1$, $-z+1$.

Reference

[1] G. M. Sheldrick, *Program for the solution and refinement of crystal structures* University of Göttingen, Germany, **1997**.

[2] C. F. Leong, B. Chan, T. B. Faust, D. M. D'Alessandro, *Chem. Sci.* **2014,** 5, 4724.

[3] J. Wu, C. Tao, Y. Li, Y. Yan, J. Li, J. Yu, *Chem. Sci.* **2014,** 5, 4237

[4] P. C. Jhang, N. T. Chuang, S. L. Wang, *Angew. Chem. Int. Ed.*, **2010,** 49, 4200; *Angew. Chem.* **2010,** 122, 4296.
